# Multi-Institutional Development and Validation of a Radiomic Model to Predict Prostate Cancer Recurrence Following Radical Prostatectomy

**DOI:** 10.3390/jcm12237322

**Published:** 2023-11-26

**Authors:** Linda My Huynh, Benjamin Bonebrake, Joshua Tran, Jacob T. Marasco, Thomas E. Ahlering, Shuo Wang, Michael J. Baine

**Affiliations:** 1 Department of Radiation Oncology, University of Nebraska Medical Center, Omaha, NE 68105, USA; linda.huynh@unmc.edu (L.M.H.); jmarasco@unmc.edu (J.T.M.); 2 Department of Urology, University of Nebraska Medical Center, Omaha, NE 68105, USA; benjamin.bonebrake@unmc.edu; 3 Department of Urology, University of California, Irvine–Health Orange, Orange, CA 92868, USA; joshuat5@hs.uci.edu (J.T.); tahlerin@uci.edu (T.E.A.); 4 Physics Department, Creighton University, Omaha, NE 68178, USA

**Keywords:** prostate cancer, radiomics, recurrence, machine learning

## Abstract

The use of multiparametric magnetic resonance imaging (mpMRI)-derived radiomics has the potential to offer noninvasive, imaging-based biomarkers for the identification of subvisual characteristics indicative of a poor oncologic outcome. The present study, therefore, seeks to develop, validate, and assess the performance of an MRI-derived radiomic model for the prediction of prostate cancer (PC) recurrence following radical prostatectomy (RP) with curative intent. mpMRI imaging was obtained from 251 patients who had undergone an RP for the treatment of localized prostate cancer across two institutions and three surgeons. All patients had a minimum of 2 years follow-up via prostate-specific antigen serum testing. Each prostate mpMRI was individually reviewed, and the prostate was delineated as a single slice (ROI) on axial T2 high-resolution image sets. A total of 924 radiomic features were extracted and tested for stability via intraclass correlation coefficient (ICC) following image normalization via histogram matching. Fourteen important and nonredundant features were found to be predictors of PC recurrence at a mean ± SD of 3.2 ± 2.2 years post-RP. Five-fold, ten-run cross-validation of the model containing these fourteen features yielded an area under the curve (AUC) of 0.89 ± 0.04 in the training set (*n* = 225). In comparison, the University of California San Fransisco Cancer of the Prostate Risk Assessment score (UCSF-CAPRA) and Memorial Sloan Kettering Cancer Center (MSKCC) Pre-Radical prostatectomy nomograms yielded AUC of 0.66 ± 0.05 and 0.67 ± 0.05, respectively (*p* < 0.01). When the radiomic model was applied to the test set (*n* = 26), AUC was 0.78; sensitivity, specificity, positive predictive value, and negative predictive value were 60%, 86%, 52%, and 89%, respectively. Accuracy in predicting PC recurrence was 81%.

## 1. Introduction

Prostate cancer (PC) recurrence following definitive therapy via radiation therapy or radical prostatectomy (RP) is common, ranging from 15% to 30% in the contemporary literature [1,2,3]. Biochemical recurrence (BCR) of PC following RP is a significant source of anxiety for patients, as it signifies the return of their PC and, often, the need for further treatment and their associated side effects. These additional treatments may include the use of androgen deprivation therapy (ADT), radiation therapy, and/or salvage surgeries—all of which may be indicated with BCR years after their initial treatment [1,2]. As such, several groups have suggested that early prediction of patients at high risk for BCR may allow for more proactive treatment regimens planned at the time of initial intervention [3,4,5]. This would not only take advantage of the synergy of combination therapy, but it would also ease patient anxiety prior to and following their PC treatment.

Unfortunately, at the time of diagnosis, clinical characteristics such as stage and grade of disease have proven to be inadequate in predicting treatment success [4]. Several tools, such as the University of California, San Francisco Cancer of the Prostate Risk Assessment (UCSF-CAPRA) [4] and the Memorial Sloan Kettering Cancer Center (MSKCC) preradical prostatectomy nomogram [5], have been proposed in an effort to model adverse outcomes following RP. However, these nomograms use only demographic and clinicopathologic features and, thus, are limited in their ability to capture adverse features and predict tumor molecular heterogeneity. Furthermore, the predictive capability of these nomograms varies widely between different cohorts [6].

In the PC clinical care pathway, multiparametric magnetic resonance imaging (mpMRI) represents a potential avenue for pretreatment prediction of PC recurrence. In 2018, the National Comprehensive Cancer Center (NCCN) guidelines recognized the utility of mpMRI in PC staging [7], and several studies have since reported improved correlation between diagnostic versus pathologic staging with the use of mpMRI [8,9,10]. While this represents a step in the right direction, the use of mpMRI remains unstandardized in the use of treatment outcome prediction and depends heavily on user interpretation and classification.

Modern advancements in machine learning methodologies have facilitated the development of radiomics, a computer-based method of extracting and quantitatively analyzing subvisual characteristics on medical imaging [11]. Radiomic features (i.e., qualities of intensity, texture, shape, or wavelet) can be extracted from a variety of medical images using advanced mathematical algorithms, aggregated into predictive models, and applied to enhance personalized therapy. In this regard, several groups have applied radiomics to screen cancer patients [12], predict adverse pathologic features [8,10,13], and grade cancers [9,14,15]. Even further, radiomic features have also been shown to be associated with tumor markers, tumor microenvironment, and other high-risk genetic features corresponding with poor oncologic outcomes [12]. As PC is highly heterogeneous, the identification of noninvasive, imaging-based biomarkers indicative of clinical outcomes can facilitate disease-tailored treatment planning. For instance, if these technologies are ultimately applied to identify men more likely to experience recurrent PC following initial treatment, these models could supplement discussions regarding treatment strategy, tolerable risks and benefits to the patient, and the need for individual and/or systemic therapies. To this end, the present study seeks to develop, validate, and assess the utility of an mpMRI-based radiomic model for the prediction of men more likely to experience PC recurrence following RP.

## 2. Materials and Methods

### 2.1. Patient Selection

From March 2012 through November 2018, mpMRI was obtained from 251 consecutive patients who had undergone a robot-assisted radical prostatectomy (RARP) for the treatment of localized PC at two institutions. All surgeries were performed by three surgeons: one with over 20 years’ experience (TA) and two with over 10 years’ experience (SB and CL). Patients were extracted from an institutional database consecutively under approved institutional review board protocol and were included in this study only if they had a pre-operative mpMRI study of the prostate and/or pelvis prior to RP. All patients had ≥2 years follow-up via serum prostate-specific antigen (PSA) levels, consistent with the average time to recurrence in RP patients. A PC recurrence was defined as a biochemical recurrence (BCR) or two consecutive blood serum prostate-specific antigen levels of 0.2 ng/mL or greater. Patients with any neoadjuvant or adjuvant treatment in conjunction with RP and/or had any PSA persistence following RP were excluded. Ethical access to follow-up information was maintained only by those with an existing clinical relationship with the subjects and/or who had existing professional responsibilities that required access to protected health information. All data, including medical imaging, were abstracted for analysis in a manner that did not include identifying information and without retaining the code to the identifiers. All data was transferred to the main institution (UNMC) for analysis after the establishment of a data use agreement and individual institutional review board approval.

Descriptive statistics were generated using Statistical Package for Social Sciences (SPSS) v28 © IBM Corporation (Armonk, NY, USA) to describe the full cohort and its subgroups, with categorical variables reported with *n*, % and continuous variables reported with mean, standard deviation for normally distributed variables, or median (interquartile range) for skewed variables. Patient demographics and clinicopathologic characteristics were compared between the training and testing datasets via Student *t*-tests for continuous variables and Pearson chi-square tests for categorical variables. Levene’s test for homogeneity of variances was used to assess for nonparametric distributions, and the Mann–Whitney U test was used for distributions violating homoscedasticity assumptions. Differences were assessed in the following subgroups: those with and without mpMRI, patients between the two institutions, and between the model training and testing sets.

#### 2.1.1. Feature Extraction and Selection

**Figure 1:**
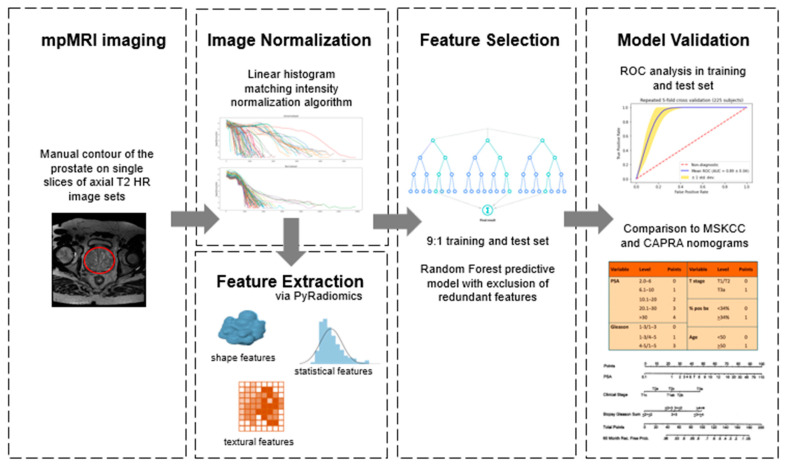
Overall workflow and radiomics pipeline.

Figure 1 illustrates the overall workflow and radiomics pipeline for the present study. Each prostate mpMRI was individually reviewed, and the prostate was delineated as a single slice (ROI) on axial T2 high-resolution image sets. One author [LMH] performed all contouring after extensive training by the senior investigator [MB]. All contours were checked for accuracy and corrected [MB]. Contouring of the region of interest was completed manually utilizing the Varian Velocity system (Varian Medical Systems, Palo Alto, CA, USA), and all investigators were blinded to the clinical outcome during this process. Bias correction was applied to all images to compensate for intensity nonuniformities using the N4 Bias Correlation approach [12]. In addition to this bias correction, we also applied a piecewise, linear histogram matching the intensity normalization algorithm (Nyul et al. methodology [16,17]) to standardize the set of MRI images prior to radiomics analysis and extracted features. All radiomic features were then extracted utilizing an open-source package on the PyRadiomics 2.0 platform, an open-source Python package for the extraction of radiomics features from medical imaging [18]. DICOM images were converted to nearly raw raster data (NRRD) file format using a batch process in the 3D Slicer 5.4 software [19] as previously described [20] prior to export.

The effects of various bin widths and resampling on the stability of extracted features were investigated [18,21,22,23]. Gray-level discretization in five different bin widths (5, 10, 25, 50, 75) was chosen and applied to the volume images and masks with original resolution. The feature extraction process was repeated with ten different parameter sets, and intraclass correlation coefficient (ICC) was utilized to quantitatively assess the stability of radiomic features against various uncertainty sources [21,22,24]. With five bin widths on images with/without resampling, the ICC of ten sets of extraction parameters categorized by feature groups was also assessed.

#### 2.1.2. Model Development

For model development and training, patients were randomly split via random number generation from 9 to 1 into training (*n* = 225) and testing (*n* = 26) datasets. After verification that there were no statistically significant differences between the training and testing datasets (described above), the training dataset was used to iteratively train and test the best parameters with a Random Forest (RF) predictive model. Redundant features were excluded with a Pearson Coefficient less than 0.4, and the most important features were selected based on five widely used classifiers (Stability Feature Selection, Random Forest, Extra Tree, Adaptive Boosting, and Extreme Gradient Boosting). From the top feature list, the most important features were selected based on the importance, redundancy, and correlation with other features after multiple iterations on five-fold, ten-run cross-validation. Based on the selected features, a predicted model was built, and a ’radiomic score’ predicting the likelihood of PC recurrence was produced for each patient.

#### 2.1.3. Model Assessment

The radiomic score predicting the likelihood of PC recurrence was assessed against the follow-up data for PC recurrence for each patient. PC recurrence was defined as biochemical recurrence, or two consecutive PSA levels greater than or equal to 0.2 ng/mL. The receiver operatorcharacteristic area under the curve analysis was utilized, and model accuracy, sensitivity, specificity, positive predictive value (PPV), and negative predictive value (NPV) were reported.

The final radiomic model was then compared to two clinical nomograms used to predict biochemical recurrence from presurgical patient characteristics: the University of California, San Fransisco—Cancer of the Prostate Risk Assessment (UCSF-CAPRA) score [4] and the Memorial Sloan Kettering Cancer Center (MSKCC) preradical prostatectomy nomogram [5]. Utilizing previously published model properties, equations, and coefficients, the predicted probability of PC recurrence was calculated for each patient from each nomogram. Predicted probabilities were aggregated via receiver operatorcharacteristic curve (ROC) analysis. The ROC area under the curve (AUC) analysis was conducted for each model and compared pairwise to the radiomic model. A *p*-value less than 0.05 was considered statistically significant for all analyses.

## 3. Results

### 3.1. Patient Demographic and Clinical Pathologic Characteristics

There were no statistically significant differences between the demographic and clinicopathologic features of the 251 included patients versus patients who had met inclusion criteria but did not have an mpMRI at the time of RP (*n* = 109). Overall, the mean ± SD age and preoperative PSA were 64.1 ± 7.3 years and 9.6 ± 9.8 ng/mL, respectively. The mean ± SD follow-up time was 3.2 ± 2.2 years. Of the 251 patients, 19.5% (49/251) experienced recurrence. All patients had follow-ups of at least two years, consistent with the average time to recurrence in PC patients following RARP.

After random characterization of 9:1 into training and test datasets, there were no statistically significant differences in any of the patient demographics or clinicopathologic features between groups (Table 1). In the training and test datasets, 19.6% (44/225) and 19.2% (5/26) patients experienced recurrence, respectively.

### 3.2. Feature Extraction and Selection

**Figure 2:**
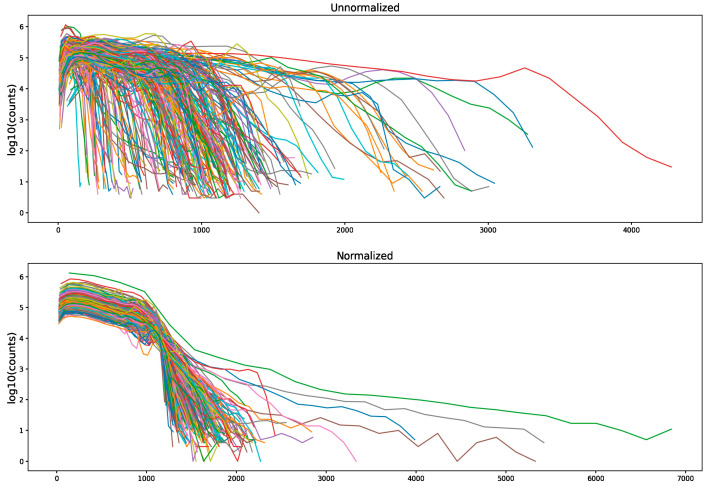
mpMRI image histograms before and after the application of intensity normalization via histogram matching.

**Figure 3:**
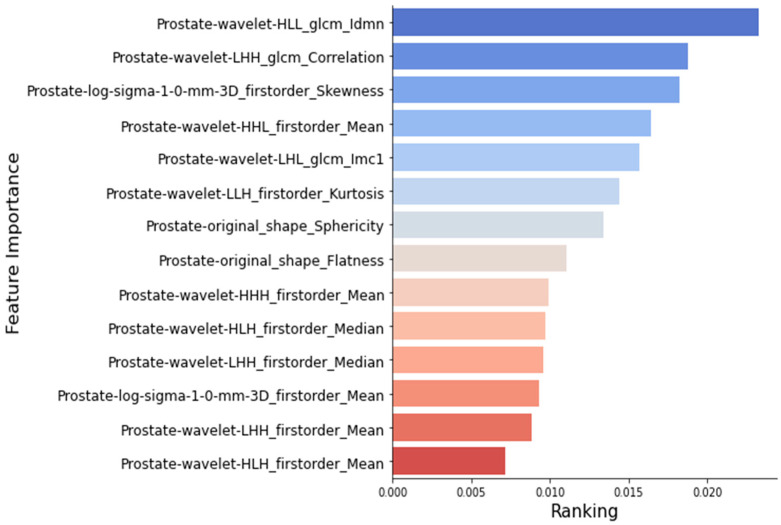
Feature importance ranking with XGBoost, Random Forest, AdaBoost, and Extra Tree.

Figure 2 depicts the image histograms before and after imaging processing via the application of intensity normalization by histogram matching. After image normalization, feature selection among 924 radiomic features yielded 137 (14.8%) stable features for assessment. Following ICC normalization, these features were reduced to fourteen stable, nonredundant, and robust features predictive of PC recurrence. Feature importance ranking is illustrated in Figure 3.

### 3.3. Radiomic Model Performance

**Figure 4:**
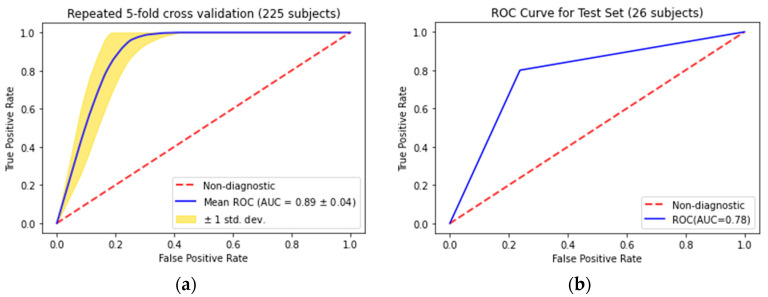
(**a**) Receiver operatorcharacteristic curve analysis of repeated 5-fold cross-validation in the model (**a**) training set and (**b**) testing set.

The fourteen most important and nonredundant radiomic features were aggregated into a model to predict PC recurrence and tested in the training dataset. Repeated five-fold cross-validation yielded a mean ROC-AUC of 0.89 ± 0.04 (Figure 4a). Sensitivity, specificity, positive predictive value, and negative predictive value in the testing set were 60%, 86%, 52%, and 90%, respectively. The accuracy of the final radiomic model was 80.88%.

Prediction of PC recurrence via the UCSF-CAPRA score and MSKCC preradical prostatectomy nomogram in the training set yielded ROC-AUC of 0.66 ± 0.05 and 0.67 ± 0.05, respectively. A pairwise comparison between the AUC for the radiomic model versus that of the UCSF-CAPRA score revealed a statistically significant improvement in the prediction of PC recurrence with the radiomic nomogram (*p* < 0.0001). Comparison with the MSKCC preradical prostatectomy also yielded a statistically significant improvement in the prediction of PC recurrence (*p* = 0.0018).

When the radiomic model was applied to the testing set, ROC-AUC analysis yielded an AUC of 0.78 (Figure 4b). Application of the UCSF-CAPRA and MSKCC preradical prostatectomy nomograms in the test set yielded ROC-AUC of 0.69 ± 0.13 and 0.75 ± 0.193, respectively.

## 4. Discussion

mpMRI-derived radiomics is an emerging field with the potential to offer noninvasive, imaging-based biomarkers for PC risk stratification and prediction of treatment response. The ability to prognosticate may not only alter risk stratification at the time of initial diagnosis but may also inform further treatment planning. The present study is unique to previous explorations utilizing radiomics as a tool for screening and/or staging during initial PC diagnosis. While these are valuable explorations, the prediction of patients at high risk for cancer recurrence is significant in its potential to enable physicians to consider proactive measures to prevent poor outcomes. The prognostication of recurrence, for example, may lead to changes in treatment strategy, the addition of adjuvant therapies, and/or the recommendation to pursue a different treatment modality. Overall, our radiomic model yielded excellent predictive capability in identifying patients at high risk for PC recurrence at a median follow-up of 3.0 (1.2) years, with no patients having less than the average two-year follow-up time for detection of a recurrence. Even further, when compared against the UCSF-CAPRA score and MSKCC preradical prostatectomy nomogram, our model yielded significantly improved prediction of PC recurrence. As we look forward to future validation and the possible creation of a radiomic-clinicopathologic nomogram, these results represent great potential in the prediction of PC recurrence.

Our goal in the use of mpMRI-derived radiomics with prognostic intent is congruent with several other groups [25,26,27,28]. A 2020 study by Bourbonne et al. was the first to report their efforts in the training and validation of an MRI-derived radiomic model for the prediction of post-RP PC biochemical recurrence (BCR) and BCR-free survival [28]. This group utilized T2WI and ADC maps of 107 patients prior to RP to extract radiomic factors correlating with BCR. Utilizing Cox regression analysis, the final radiomic model yielded a high negative predictive value of 96%, i.e., a reliable indicator of patients who were at very low risk of recurrence following RP. These results were echoed by three other groups [22,25,26]. First, Yan et al. reported similar models with ROC-AUCs between 0.84 and 0.88 for the prediction of 3-year BCR [22]. Similarly, Shiradkar and colleagues developed an MRI-derived radiomic model [25] that was significantly and independently correlated with 3-year BCR in Cox proportional hazards regression modeling (HR: 2.91, 95%CI: 1.45–11.51, *p* = 0.02). Finally, Li. and colleagues [26] found that integration of clinicopathologic features with radiomic analysis yielded a model also independently associated with recurrence following RP (HR:7.01, 95%CI:1.21–40.68, *p* < 0.05).

Of four above-mentioned studies, the present study is the second largest series overall (second to Yan et al. [22]) and includes the largest number of patients in the model training set. This increase in sample size not only translates to improved statistical power but it also introduces significant heterogeneity to the patient cohort. As such, it is important to note that the present cohort underwent RARP by three different surgeons across two institutions, each surgeon with differing years of experience, different preoperative protocols, and varying use of mpMRI in PC staging. Moreover, of these three surgeons, one also operated based on a referral system—i.e., most patients included from his cohort were diagnosed and imaged off-site. This significantly increased the number of different imaging protocols throughout the dataset, with mpMRI imaging coming from several institutions and transferred via different electronic health record systems. However, despite the multitude of inter- and intra-cohort differences, the imaging normalization techniques employed herein allowed the generation of a strong radiomic model for prediction in all patients. In this regard, to our knowledge, the present study is also the only investigation to compare a radiomic model’s performance to currently established predictive models in clinical use. This, combined with the image normalization techniques and expanded sample size of the present study, allows for increased generalizability and applicability of our model to external institutions in future explorations.

While the result of this investigation is promising, radiomics in PC prognostication is still in its infancy, and there are several limitations to the present study. First, methodological modifications to the radiomics pipeline must be intentional, and results must be considered within this context. Given that radiomic models are highly sensitive to modifications to the procedures of image segmentation, feature extraction, and feature selection, investigations regarding radiomic feature variability, robustness of available datasets, and reproducibility in multiple cohorts are required prior to consideration for clinical integration [29,30]. While the present study optimizes the feature selection pathway to include morphologic features, a correlation of these radiomic features must be established with clinicopathologic characteristics prior to expanding the radiomic model to more institutions for external validation. Similarly, the limited sample size in our testing cohort and differences in institutional procedures continue to be challenging as we continue to explore applications to a larger patient cohort. While standardized feature extraction and histogram matching in our cohort were adequate in controlling for most of these differences, this will have to be even further confirmed.

As the field of radiomics continues to grow and develop, intersectionality with histology [31,32], pathology [33,34], and genomics [35,36] offers high potential for biologic validation and improved clinical interpretability of radiomic models. Correlation with local pathologic analysis, for example, can provide a direct comparison of quantitative pathologic features to explain structural characteristics underlying radiologic textures. Even further, correlation with genomic data can provide a link to the molecular pathways underlying tumor biologic characteristics. These explorations would not only facilitate a comprehensive understanding of the interplay between macroscopic imaging features and microscopic tissue properties, but they can also facilitate the translation of radiomic findings into clinically actionable insights.

## 5. Conclusions

Given that radiomics has the potential to facilitate noninvasive characterization of tumor heterogeneity, the present study offers a multi-institutional development and validation of an mpMRI-derived radiomic model in predicting PC recurrence. In our cohort of 251 prostate cancer patients, radiomic analysis yielded fourteen radiomic features significantly associated with BCR following definitive treatment. When these features were aggregated into a radiomic signature, the model predicted PC recurrence with 81% accuracy. Furthermore, when the predictive capability of this radiomic model was compared with the clinical standard of the UCSF-CAPRA score and MSKCC preradical prostatectomy nomogram, the radiomic model illustrated significantly higher ROC-AUC. As external validations and expansion of the current study are considered, future projects will aim to incorporate patient demographics and disease characteristics to further improve the model’s sensitivity and PPV. Prior to consideration for clinical integration, however, these radiomic models must be biologically validated.

## Figures and Tables

**Table 1:** Demographic and Clinicopathologic Characteristics.

	Training Dataset		Testing Dataset		
	*n* = 225		*n* = 26		
	Mean	SD	Mean	SD	*p*
Age, years	63.4	7.2	64.5	6.9	0.460
Preoperative PSA, ng/mL	9.8	10.2	8.0	5.1	0.368
Follow-up Time, years	3.2	2.2	3.2	1.9	0.850
	*n*	%	*n*	%	*p*
Clinical Tumor Stage					0.315
1	141	66.5	18	69.2	
2	60	28.3	5	19.2	
3	11	5.2	3	11.5	
Missing	13	5.8	0	0.0	
Preoperative Gleason Grade Group					0.096
1	32	14.7	1	3.8	
2	91	41.7	11	42.3	
3	42	19.3	3	11.5	
4	30	13.8	4	15.4	
5	23	10.6	7	26.9	
Missing	7	3.1	0	0.0	
Pathologic Tumor Stage					0.814
2a	55	24.4	8	30.8	
2b	56	24.9	7	26.9	
2c	21	9.3	1	3.8	
3a	64	28.4	8	30.8	
3b	24	10.7	1	3.8	
4	1	0.4	0	0.0	
Pathologic Gleason Grade Group					0.528
1	21	9.5	2	8.0	
2	110	49.8	9	36.0	
3	50	22.6	8	32.0	
4	14	6.3	1	4.0	
5	26	11.8	5	20.0	
Seminal Vesicle Invasion	22	9.8	1	3.8	0.564
Extraprostatic Extension	76	33.8	9	34.6	1.000
Lymph Node Invasion	5	2.2	2	7.7	0.157
Positive Margins	60	27.3	66	24.0	0.467
Recurrence	44	19.6	5	19.2	1.000

## Data Availability

Data are contained within the article.

## References

[1] Pound C.R., Partin A.W., Eisenberger M.A., Chan D.W., Pearson J.D., Walsh P.C. (1999). Natural History of Progression after PSA Elevation Following Radical Prostatectomy. JAMA.

[2] Boorjian S.A., Thompson R.H., Tollefson M.K., Rangel L.J., Bergstralh E.J., Blute M.L., Karnes R.J. (2011). Long-Term Risk of Clinical Progression after Biochemical Recurrence Following Radical Prostatectomy: The Impact of Time from Surgery to Recurrence. Eur. Urol..

[3] Novara G., Ficarra V., Mocellin S., Ahlering T.E., Carroll P.R., Graefen M., Guazzoni G., Menon M., Patel V.R., Shariat S.F. (2012). Systematic Review and Meta-Analysis of Studies Reporting Oncologic Outcome after Robot-Assisted Radical Prostatectomy. Eur. Urol..

[4] Zelic R., Garmo H., Zugna D., Stattin P., Richiardi L., Akre O., Pettersson A. (2020). Predicting Prostate Cancer Death with Different Pretreatment Risk Stratification Tools: A Head-to-Head Comparison in a Nationwide Cohort Study. Eur. Urol..

[5] Cagiannos I., Karakiewicz P., Eastham J.A., Ohori M., Rabbani F., Gerigk C., Reuter V., Graefen M., Hammerer P.G., Erbersdobler A. (2003). A Preoperative Nomogram Identifying Decreased Risk of Positive Pelvic Lymph Nodes in Patients With Prostate Cancer. J. Urol..

[6] Cimino S., Reale G., Castelli T., Favilla V., Giardina R., Russo G.I., Privitera S., Morgia G. (2017). Comparison between Briganti, Partin and MSKCC Tools in Predicting Positive Lymph Nodes in Prostate Cancer: A Systematic Review and Meta-Analysis. Scand. J. Urol..

[7] Carroll P.H., Mohler J.L. (2018). NCCN Guidelines Updates: Prostate Cancer and Prostate Cancer Early Detection. J. Natl. Compr. Canc. Netw..

[8] He D., Wang X., Fu C., Wei X., Bao J., Ji X., Bai H., Xia W., Gao X., Huang Y. (2021). MRI-Based Radiomics Models to Assess Prostate Cancer, Extracapsular Extension and Positive Surgical Margins. Cancer Imaging.

[9] Chaddad A., Niazi T., Probst S., Bladou F., Anidjar M., Bahoric B. (2018). Predicting Gleason Score of Prostate Cancer Patients Using Radiomic Analysis. Front. Oncol..

[10] Xu L., Zhang G., Zhao L., Mao L., Li X., Yan W., Xiao Y., Lei J., Sun H., Jin Z. (2020). Radiomics Based on Multiparametric Magnetic Resonance Imaging to Predict Extraprostatic Extension of Prostate Cancer. Front. Oncol..

[11] Zhang Y.-P., Zhang X.-Y., Cheng Y.-T., Li B., Teng X.-Z., Zhang J., Lam S., Zhou T., Ma Z.-R., Sheng J.-B. (2023). Artificial Intelligence-Driven Radiomics Study in Cancer: The Role of Feature Engineering and Modeling. Mil. Med. Res..

[12] Castaldo R., Cavaliere C., Soricelli A., Salvatore M., Pecchia L., Franzese M. (2021). Radiomic and Genomic Machine Learning Method Performance for Prostate Cancer Diagnosis: Systematic Literature Review. J. Med. Internet Res..

[13] Ma S., Xie H., Wang H., Yang J., Han C., Wang X., Zhang X. (2020). Preoperative Prediction of Extracapsular Extension: Radiomics Signature Based on Magnetic Resonance Imaging to Stage Prostate Cancer. Mol. Imaging Biol..

[14] Liu H., Liu S., Guo D., Zheng Y., Tang P., Dan G. (2019). Original Intensity Preserved Inhomogeneity Correction and Segmentation for Liver Magnetic Resonance Imaging. Biomed. Signal Process. Control.

[15] Penzias G., Singanamalli A., Elliott R., Gollamudi J., Shih N., Feldman M., Stricker P.D., Delprado W., Tiwari S., Böhm M. (2018). Identifying the Morphologic Basis for Radiomic Features in Distinguishing Different Gleason Grades of Prostate Cancer on MRI: Preliminary Findings. PLoS ONE.

[16] Nyúl L.G., Udupa J.K., Zhang X. (2000). New Variants of a Method of MRI Scale Standardization. IEEE Trans. Med. Imaging.

[17] Single-Cell Spatial Proteomic Revelations on the Multiparametric MRI Heterogeneity of Clinically Significant Prostate Cancer|Clinical Cancer Research|American Association for Cancer Research. https://aacrjournals.org/clincancerres/article/27/12/3478/671458/Single-cell-Spatial-Proteomic-Revelations-on-the.

[18] Van Griethuysen J.J.M., Fedorov A., Parmar C., Hosny A., Aucoin N., Narayan V., Beets-Tan R.G.H., Fillion-Robin J.-C., Pieper S., Aerts H.J.W.L. (2017). Computational Radiomics System to Decode the Radiographic Phenotype. Cancer Res..

[19] Pieper S., Lorensen B., Schroeder W., Kikinis R. The NA-MIC Kit: ITK, VTK, Pipelines, Grids and 3D Slicer as An Open Platform for the Medical Image Computing Community. Proceedings of the 3rd IEEE International Symposium on Biomedical Imaging: Macro to Nano.

[20] Wang S., Lin C., Kolomaya A., Ostdiek-Wille G.P., Wong J., Cheng X., Lei Y., Liu C. (2022). Compute Tomography Radiomics Analysis on Whole Pancreas Between Healthy Individual and Pancreatic Ductal Adenocarcinoma Patients: Uncertainty Analysis and Predictive Modeling. Technol. Cancer Res. Treat..

[21] Rdrr.io. Irr: Various Coefficients of Interrater Reliability and Agreement Version 0.84.1 from CRAN. https://rdrr.io/cran/irr/.

[22] Yan Y., Shao L., Liu Z., He W., Yang G., Liu J., Xia H., Zhang Y., Chen H., Liu C. (2021). Deep Learning with Quantitative Features of Magnetic Resonance Images to Predict Biochemical Recurrence of Radical Prostatectomy: A Multi-Center Study. Cancers.

[23] Larue R.T.H.M., van Timmeren J.E., de Jong E.E.C., Feliciani G., Leijenaar R.T.H., Schreurs W.M.J., Sosef M.N., Raat F.H.P.J., van der Zande F.H.R., Das M. (2017). Influence of Gray Level Discretization on Radiomic Feature Stability for Different CT Scanners, Tube Currents and Slice Thicknesses: A Comprehensive Phantom Study. Acta Oncol..

[24] Koo T.K., Li M.Y. (2016). A Guideline of Selecting and Reporting Intraclass Correlation Coefficients for Reliability Research. J. Chiropr. Med..

[25] Shiradkar R., Ghose S., Mahran A., Li L., Hubbard I., Fu P., Tirumani S.H., Ponsky L., Purysko A., Madabhushi A. (2022). Prostate Surface Distension and Tumor Texture Descriptors From Pre-Treatment MRI Are Associated With Biochemical Recurrence Following Radical Prostatectomy: Preliminary Findings. Front. Oncol..

[26] Li L., Shiradkar R., Leo P., Algohary A., Fu P., Tirumani S.H., Mahran A., Buzzy C., Obmann V.C., Mansoori B. (2021). A Novel Imaging Based Nomogram for Predicting Post-Surgical Biochemical Recurrence and Adverse Pathology of Prostate Cancer from Pre-Operative Bi-Parametric MRI. eBioMedicine.

[27] Dou T.H., Coroller T.P., van Griethuysen J.J.M., Mak R.H., Aerts H.J.W.L. (2018). Peritumoral Radiomics Features Predict Distant Metastasis in Locally Advanced NSCLC. PLoS ONE.

[28] Bourbonne V., Fournier G., Vallières M., Lucia F., Doucet L., Tissot V., Cuvelier G., Hue S., Le Penn Du H., Perdriel L. (2020). External Validation of an MRI-Derived Radiomics Model to Predict Biochemical Recurrence after Surgery for High-Risk Prostate Cancer. Cancers.

[29] Hatt M., Laurent B., Ouahabi A., Fayad H., Tan S., Li L., Lu W., Jaouen V., Tauber C., Czakon J. (2018). The First MICCAI Challenge on PET Tumor Segmentation. Med. Image Anal..

[30] Zwanenburg A., Löck S. (2018). Why Validation of Prognostic Models Matters?. Radiother. Oncol..

[31] Geady C., Keller H., Siddiqui I., Bilkey J., Dhani N.C., Jaffray D.A. (2020). Bridging the gap between micro- and macro-scales in medical imaging with textural analysis—A biological basis for CT radiomics classifiers?. Phys. Med..

[32] Bobholz S.A., Lowman A.K., Barrington A., Brehler M., McGarry S., Cochran E.J., Connelly J., Mueller W.M., Agarwal M., O’Neill D. (2020). Radiomic Features of Multiparametric MRI Present Stable Associations With Analogous Histological Features in Patients With Brain Cancer. Tomography.

[33] Saltz J., Almeida J., Gao Y., Sharma A., Bremer E., DiPrima T., Saltz M., Kalpathy-Cramer J., Kurc T. (2017). Towards Generation, Management, and Exploration of Combined Radiomics and Pathomics Datasets for Cancer Research. AMIA Jt. Summits Transl. Sci. Proc..

[34] Familiar A.M., Mahtabfar A., Fathi Kazerooni A., Kiani M., Vossough A., Viaene A., Storm P.B., Resnick A.C., Nabavizadeh A. (2023). Radio-pathomic approaches in pediatric neuro-oncology: Opportunities and challenges. Neurooncol. Adv..

[35] Ismail M., Craig S., Ahmed R., de Blank P., Tiwari P. (2023). Opportunities and Advances in Radiomics and Radiogenomics for Pediatric Medulloblastoma Tumors. Diagnostics.

[36] Skingen V.E., Hompland T., Fjeldbo C.S., Salberg U.B., Helgeland H., Ragnum H.B., Aarnes E.K., Vlatkovic L., Hole K.H., Seierstad T. (2023). Prostate cancer radiogenomics reveals proliferative gene expression programs associated with distinct MRI-based hypoxia levels. Radiother. Oncol..

